# Adult attachment and hypersexuality: links to couple and sexual satisfaction in a Mexican sample

**DOI:** 10.3389/fpsyg.2026.1911233

**Published:** 2026-10-05

**Authors:** Diana Mejía-Cruz, Laurent Avila-Chauvet, Luis Villalobos-Gallegos

**Affiliations:** 1 Psychology Department, Sonora Institute of Technology, Ciudad Obregón, Mexico; 2 School of Medicine and Psychology, Autonomous University of Baja California, Tijuana, Mexico

**Keywords:** attachment styles, couple satisfaction, hypersexuality, psychological distress, sexual satisfaction

## Abstract

**Introduction:**

Previous research has linked anxious and avoidant attachment with emotional dysregulation and hypersexuality-related symptoms and has documented associations between risky sexual behaviors and adverse health outcomes. However, the relationships of sexual and couple satisfaction with hypersexuality remain comparatively underexplored, particularly in Mexican populations.

**Objective:**

This study examined the associations among attachment dimensions, hypersexuality-related symptoms, risky sexual behaviors, psychological distress, sexual satisfaction, and couple satisfaction in a sample of Mexican adults.

**Methods:**

A total of 214 participants completed the Sexual Addiction Screening Test-Revised (SAST-R), Couple Satisfaction Index-4 (CSI-4), Experiences in Close Relationships Scale (ECR-8), Symptom Checklist-90 (SCL-90), and measures of risky sexual behaviors. Data were analyzed using Classification and Regression Trees (CART), logistic regression, correlational analyses, and Mann–Whitney *U* tests.

**Results:**

Higher SAST-R scores were associated with several risky sexual behaviors, including a greater number of sexual partners, substance use during sexual activity, and lack of sexual protection. Psychological distress and risky sexual behaviors also contributed to the exploratory classification of participants according to SAST-R scores. Participants with higher SAST-R scores showed descriptively lower sexual satisfaction; however, these differences were not statistically significant. Contrary to expectations, neither attachment anxiety nor attachment avoidance was significantly associated with SAST-R scores. Nevertheless, attachment avoidance was negatively associated with couple and sexual satisfaction and was associated with age at sexual debut.

**Discussion and conclusion:**

The findings suggest that psychological distress and risky sexual behaviors may be more closely associated with hypersexuality-related symptoms than attachment dimensions in the present sample. In contrast, attachment avoidance appears to be associated with selected aspects of sexual and relational functioning, particularly sexual and couple satisfaction. These findings underscore the importance of distinguishing correlates of hypersexuality-related symptoms from broader relational functioning and suggest that prevention and intervention efforts should prioritize psychological distress, sexual-health risk reduction, and promoting secure attachment patterns.

## 1 Introduction

Attachment theory, initially formulated to explain the emotional bond between infants and caregivers, has since been expanded to include close relationships in adulthood. Bowlby (1982) defined attachment as the infant's tendency to seek proximity to a primary caregiver during childhood. Since Bowlby's (1982) theory of attachment, researchers have explored whether the three attachment styles identified in childhood, secure, avoidant, and anxious/ambivalent, persist into adulthood. Hazan and Shaver (1987) provided evidence that adults exhibit these same attachment styles, which are associated with distinct beliefs about the nature of romantic love, the availability and trustworthiness of partners, and their own worthiness of love.

Attachment and sexual mating, while distinct behavioral systems with different evolutionary functions, often overlap in romantic relationships (Birnbaum, 2010). Although their behavioral manifestations may occur independently, romantic partners typically serve simultaneously as sexual partners and attachment figures. According to Birnbaum (2010), individual differences in the functioning of the sexual system can be conceptualized in terms of anxious hyperactivation or avoidant deactivation of the sexual response. For instance, studies by Birnbaum et al. (2011, 2014) demonstrated the role of the sexual system in regulating reactions to attachment-related stress. These studies investigated the effects of relationship threats on sexual desire and the motivations for engaging in sexual activity. Relationship threats were found to prompt both enhancement motives and relationship-based motives, such as engaging in sex to nurture a partner, suggesting that individuals use sex to enhance physical or emotional pleasure and to repair threatened relationships. Sexual comfort, which represents the optimal functioning of the sexual system, is in the region where both anxious hyperactivation and avoidant deactivation are low. This state is characterized by confidence in one's sexual desirability and abilities. Conversely, higher levels of sexual hyperactivation and/or deactivation reflect deviations from this optimal region, rendering sex a source of distress rather than pleasure.

### 1.1 Attachment, hypersexuality, and sexual risk behaviors in adults

In this context, insecure adult attachment has been associated with difficulties in intimate relationships and with dysregulated sexual behavior. Several studies have reported associations between attachment insecurity, particularly attachment anxiety, and measures conceptualized as sexual addiction or hypersexuality, although avoidant attachment has also been implicated (Gori and Topino, 2024; Ciocca et al., 2021; Kotera and Rhodes, 2019; Gilliland et al., 2015; Zapf et al., 2008; Weinstein et al., 2015). However, hypersexuality, sexual addiction, and compulsive sexual behavior should not be treated as interchangeable diagnostic constructs. Rather, they represent overlapping conceptualizations of persistent or difficult-to-control sexual thoughts, urges, and behaviors associated with distress, impaired control, or adverse consequences. Karila et al. (2014) noted substantial overlap between sexual addiction and the proposed construct of hypersexual disorder, while also emphasizing inconsistencies in their definitions, theoretical models, and assessment. Similarly, Walton et al. (2017) conceptualized hypersexuality as a heterogeneous phenomenon that may arise through different pathways, including heightened sexual excitation and impulsivity, the regulation of dysphoric affect and stress, and difficulties in interpersonal connectedness. Within the sex-addiction model specifically, trauma, adverse childhood experiences, attachment disturbances, impaired impulse control, shame, and mood dysregulation have been proposed as possible underlying factors; however, this addiction framework represents only one theoretical account of hypersexuality rather than an established explanation of the construct (Walton et al., 2017). More recently, compulsive sexual behavior (CSB) has increasingly been used as an umbrella term for persistent, dysregulated, or out-of-control sexual behavior, encompassing much of the earlier literature described as sexual addiction, hypersexuality, or sexual compulsivity, while debate continues regarding whether these behaviors are best conceptualized as addictive, impulsive, or compulsive processes (Grubbs et al., 2020). Accordingly, in the present study, hypersexuality is used descriptively to refer to elevated self-reported symptoms of problematic sexual behavior rather than as synonymous with sexual addiction or as a formal clinical diagnosis.

Understanding the relationship between attachment and hypersexuality is crucial in the current study. Insecure attachment has been associated with chemsex, or chemical sex, which involves the use of drugs to reduce inhibition and enhance sexual pleasure (Blanc et al., 2023). In a study conducted with an adolescent sample, higher levels of attachment anxiety and avoidance were found to predict less healthy sexual attitudes. Moreover, elevated levels of attachment avoidance have been linked to increased sexual risk-taking behaviors, such as a higher number of sexual partners and low condom use, particularly among non-virgin men (McElwain et al., 2015). Similarly, a study of adults in their 30s found that insecure attachment predicts an earlier sexual debut, a higher number of both casual and committed sexual partners, and an increased likelihood of remaining single (Busby et al., 2020). These findings align with evidence suggesting that hypersexuality is primarily rooted in attachment processes. Traumatic experiences, particularly childhood sexual abuse, and symptoms of depression may further mediate hypersexual behavior (Ciocca et al., 2021; Miner et al., 2016a,b; Miner et al., 2010). The adverse consequences of hypersexuality can cause significant impairment in daily functioning and are associated with depressed mood and anxiety, which particularly impact close and romantic relationships (Kafka, 2010; Dhuffar et al., 2015).

Secure attachment has been associated with a positive self-concept, greater trust in others, willingness to seek support when needed, and more adaptive emotional regulation, all of which may contribute to psychological wellbeing and healthier interpersonal relationships (Shelton and Wang, 2018). In contrast, insecure attachment has been associated with difficulties in emotional and interpersonal regulation and with greater psychological distress (Altan-Atalay and Sohtorik Ilkmen, 2020; Corcoran and McNulty, 2018; Rettenberger et al., 2016), lower sexual satisfaction (Butzer and Campbell, 2008; Lafortune et al., 2022), and lower relationship and marital satisfaction (Butzer and Campbell, 2008). Consistent with these findings, attachment insecurity has also been associated with lower scores on measures of couple satisfaction and with relationship difficulties, including infidelity (Díaz-Pichardo et al., 2020). A meta-analysis of 132 studies further demonstrated that higher levels of attachment anxiety and avoidance were associated with lower relationship satisfaction (Candel and Turliuc, 2019). Accordingly, attachment processes have become relevant targets in couple-based interventions aimed at reducing relational distress and persistent conflict (Feeney and Karantzas, 2017; Morgis et al., 2019; Wiebe et al., 2019).

Additionally, insecure attachment has been linked to various other factors, including low self-esteem (Shen et al., 2021), increased depressive symptoms (Ciocca et al., 2021), and reduced interpersonal trust (Welch and Houser, 2010). Previous studies have explored the mediating role of variables such as financial status and motivation to forgive in understanding the correlation between secure attachment and couple satisfaction, confirming both the direct and indirect relationships among these variables (Guzmán-González et al., 2020; Li et al., 2021). In contrast, the mediation variables for understanding hypersexuality have focused on the roles of shame, depression, and loneliness symptoms in individuals experiencing post-traumatic stress disorder (PTSD) (Fontanesi et al., 2021). In some cases, these symptoms not only mediate but also directly predict hypersexual behavior and low life satisfaction (Dhuffar et al., 2015). This relationship between PTSD and hypersexual behavior is strongly associated with evidence that psychological and sexual abuse serve as developmental antecedents of hypersexuality (Knight and Du, 2021).

The stability of attachment styles from childhood to adulthood is weakly associated, suggesting that earlier experiences do not necessarily determine adult outcomes (Corcoran and McNulty, 2018; Fraley and Roisman, 2019). However, according to some longitudinal studies, variations in infant–caregiver attachment form the foundation for later individual differences in personality (Sroufe, 2005). In a longitudinal study that followed participants over 30 years, Young et al. (2019) found that secure attachment at 18 months predicted scores on the Big Five personality traits at age 32, particularly focusing on the traits that constitute the meta-trait known as stability (high agreeableness, high conscientiousness, and low neuroticism).

In this framework, certain personality traits become essential in the context of romantic relationships. Agreeableness, for example, reflects a tendency toward stable relationships, while low neuroticism suggests emotional stability, both of which are critical for romantic interactions (DeYoung, 2006). Previous studies have found that lower levels of agreeableness, conscientiousness, and openness, alongside higher levels of neuroticism, are associated with hypersexuality (Carvalho et al., 2015; Dini et al., 2018; Mejía Cruz et al., 2023; Rettenberger et al., 2016).

### 1.2 Defining the scope: aims and hypotheses

Despite substantial evidence linking attachment insecurity separately with psychological distress, sexual satisfaction, and relationship satisfaction, less is known about how these relational and psychological dimensions are jointly associated with hypersexuality. Previous research has more often examined individual correlates of hypersexuality than the simultaneous relationships among attachment dimensions, sexual satisfaction, couple satisfaction, psychological distress, and sexual-behavior characteristics within the same sample. This distinction is important because hypersexuality occurs within a broader interpersonal and psychological context, and examining these domains concurrently may help clarify whether relational variables such as attachment and couple satisfaction are associated with hypersexuality beyond the psychological distress and sexual-behavior characteristics commonly examined in this literature. The present study addresses this gap by examining these constructs simultaneously in a sample of Mexican adults, a population that remains comparatively underrepresented in research on hypersexuality and its relational correlates. We propose an initial correlational study among these variables to provide evidence within the Mexican population.

Research on sexual behavior among adolescents and adults in Mexico has primarily focused on sexual health and risk-related outcomes, including HIV/AIDS and other consequences of risky sexual behavior (Castillo-Arcos et al., 2016), individual factors such as impulsivity (Espinosa-Hernández et al., 2021), and the role of family processes and cultural values in sexual behavior (Espinosa-Hernández et al., 2020; Velazquez et al., 2023). In particular, research with Mexican adolescents indicates that sexuality is embedded within a broader sociocultural and familial context in which values such as parental monitoring, and gender-specific expectations are associated with sexual attitudes, intentions, and behaviors (Velazquez et al., 2023). Moreover, sexual socialization in the Mexican context may differ by gender, with greater sexual permissiveness traditionally afforded to males and greater restrictions and expectations regarding sexual behavior placed on females (Velazquez et al., 2023). Such sociocultural processes may shape how individuals experience and report sexual behavior, relationship satisfaction, and attachment-related interpersonal difficulties. Although some research has examined attachment-related processes in Mexican populations, such as disorganized attachment in relation to psychosis-risk symptoms (Sheinbaum et al., 2024), evidence concerning attachment in the context of hypersexuality and intimate relationship functioning remains limited. Thus, examining hypersexuality, attachment dimensions, sexual satisfaction, and couple satisfaction in a Mexican sample may extend the predominantly individual- and risk-focused literature by situating these constructs within a sociocultural context in which family values, gender norms, and relational expectations may be particularly salient.

This study examines the associations between insecure attachment, specifically, attachment anxiety and avoidance, and risky sexual behaviors, including multiple sexual partners, unprotected sexual activity, and substance use during sexual encounters. It also investigates relationships among attachment styles, hypersexuality, psychological distress, sexual satisfaction, and couple satisfaction. The key research question asks whether hypersexuality stems from deficiencies in maintaining safe interpersonal relationships, essential for healthy sexuality and relationship satisfaction. The study hypothesizes a positive correlation between attachment anxiety/avoidance and hypersexuality, expecting that individuals with higher hypersexual behaviors will engage in more sexual risk behaviors and experience increased psychological distress, while also showing lower couple and sexual satisfaction.

## 2 Materials and methods

We utilized a cross-sectional design, which was complemented by a snowball sampling strategy. All participants were residents of urban areas within cities in the north of México, aged between 18 and 50 years, and possessed adequate literacy skills.

### 2.1 Participants

We enrolled 214 adult participants from a pool of 307 individuals who completed all psychological measures. Of these, 104 identified as men (mean age: *M* = 21.63, SD = 5.66), 103 as women (mean age: *M* = 21.95, SD = 5.55), four identified with a non-binary gender (mean age: *M* = 21.75, SD = 5.67), and three did not specify their gender (mean age: *M* = 22.33, SD = 8.38). In terms of sexual orientation, 73.3% of the sample identified as heterosexual, 0.4% as gay, 3.2 as lesbian, 10.7% as bisexual, 4.2% were unsure of their orientation, and 7.9% did not respond to the question. The average number of years of education was *M* = 14.20 (SD = 3.93), with a mean monthly income of *M* = 164.54 USD (SD = 397.48). The marital status of the sample was as follows: 1.8% were married, 97% were single, and 1.2% were in a concubinage arrangement. Among the single participants, 62.1% reported being in a serious and exclusive relationship, 25.5% were not engaged in any form of romantic or sexual relationship, and 9.5% were engaged in non-serious relationships with multiple partners, involving both romantic and sexual experiences.

Participants reported no history of neurological disorders, substance use disorders, or any psychiatric diagnoses. This information was collected from their responses after providing informed consent. All participants provided written informed consent in accordance with the Declaration of Helsinki and did not receive financial compensation for their participation. Participants affirmed their lack of awareness of the study hypotheses. The study protocol received approval from the Institutional Review Board of the Sonora Institute of Technology (ID 178).

### 2.2 Measures

We developed a website to host online surveys, which were designed to collect information, including demographic characteristics (e.g., age, years of education, gender, income, neurological and psychiatric diagnoses) and psychological measures. To minimize participant withdrawal from the study, we distributed 232 items across 30 pages, with an anticipated completion time of 30 to 40 min.

*Sexual behavior*. We examined several key factors, including: the age at which individuals engage in sexual activity, the number of sexual partners they have had, their utilization of contraceptives, the consumption of alcohol during sexual encounters, and the use of drugs during such interactions.

*Sexual addiction (SAST-R) (Carnes et al., 2010)*. The latest iteration of the Sexual Addiction Screening Test-Revised includes 20 fundamental items with binary response options, which are self-administered (e.g., Do you often find yourself preoccupied with sexual thoughts? YES/NO). This 20-item version was selected for its applicability across different sexual orientations, making it inclusive rather than exclusive to heterosexual individuals. The confirmatory factor analysis (CFA) employed a five-factor model with a second-order factor, which demonstrated the most robust fit, and the reliability analysis indicated acceptable internal consistency (Cronbach's alpha = 0.80) (Mejía Cruz et al., 2023). This scale categorizes sexual addiction into five dimensions: (1) affect disturbance (items 4, 5, 11, 13, and 14), characterized by a significant decrease in mood associated with sexual behaviors; (2) relationship disturbance (items 6, 8, and 16), One's own sexual behavior is affecting relationships with friends, family, and work; 3) preoccupation (items 3, 18, 19, and 20), defined by obsessive sexual thoughts; (4) loss of control (items 10, 12, 15, and 17), where individuals are unable to regulate their sexual behaviors despite adverse consequences; (5) associated features (items 1, 2, 7, and 9), which include traumatic sexual experiences. We used the total score as a unit of analysis. Higher scores on the SAST-R indicate a greater degree of hypersexuality. It is important to note that SAST-R does not assess specific sexual behavior variables such as number of sexual partners, contraceptive use, or alcohol and drug consumption during sexual activity. Therefore, these variables were assessed separately as part of the demo-graphic and sexual behavior questionnaire.

*Experiences in close relationships-revised (ECR-8):* The ECR-8 consists of 8 items assessing adult attachment dimensions: anxiety and Avoidance. Items are rated on a 7-point Likert scale (1 = strongly disagree, 7 = strongly agree) (e.g., I worry that romantic partners won't care about me as much as I care about them) (Fraley et al., 2000). Each subscale contains four items, with higher scores indicating greater attachment anxiety or avoidance. In a Latino college student sample, internal consistency was α = 0.93 for Anxiety and α = 0.94 for Avoidance (Müller et al., 2024; Shelton and Wang, 2018; Wei et al., 2007). Psychometric properties in this study were acceptable (Ω = 0.99 for Anxiety, Ω = 0.99 for Avoidance).

*Couple satisfaction index (CSI-4):* the CSI-4 consists of four items assessing relationship satisfaction on a scale from 0 (Always disagree/Never/Extremely unhappy) to 5 or 6 (Always agree/Perfect/All the time/Completely true; e.g., Please indicate the degree of happiness in your relationship). Higher scores indicate greater satisfaction. The CSI-4, a brief version, demonstrated strong psychometric properties (α = 0.872) (El Frenn et al., 2022; Díaz-Pichardo et al., 2020; Forouzesh Yekta et al., 2017; Funk and Rogge, 2007; Hayee and Kamal, 2024; Kim et al., 2022). In this study, psychometric properties were acceptable (Ω = 0.93).

*The new sexual satisfaction scale-short (NSSS-S)*: the NSSS-S is a 12-item instrument designed to assess global sexual satisfaction, irrespective of gender, sexual orientation, or relationship status. Responses are recorded on a 5-point Likert scale (1 = not at all satisfied, 5 = extremely satisfied; e.g., the balance between what I give and receive in sex). Items 1–6 constitute the individual sexual satisfaction factor, while items 7–12 constitute the interpersonal factor. The instrument demonstrated strong internal reliability, with a Cronbach's alpha coefficient of 0.92 for the overall scale,0.88 for the individual subscale, and 0.87 for the interpersonal subscale (Strizzi et al., 2016; Štulhofer et al., 2010). Each subscale is analyzed as distinct units of analysis.

*Symptom checklist 90 (SCL-90) (Derogatis et al., 1976):* the SCL-90 is a psychometric instrument consisting of 90 items, each rated on a Likert scale ranging from 0 “not at all” to 4 “extremely” (e.g., How much were you bothered by: feeling easily annoyed or irritated). It assesses the level of psychological distress experienced by the respondent in the period from the day of evaluation up to 1 week prior. For seven of the nine dimensions, as well as the Global Severity Index (GSI), Cronbach's alpha demonstrated internal consistency values greater than 0.7. Meanwhile, the remaining dimensions registered scores above 0.66 (Cruz Fuentes et al., 2005). The analysis was conducted using the total score of the instrument, and each subscale was also evaluated as a separate unit.

### 2.3 General procedures

The translation and cultural adaptation of the Couple Satisfaction Index-4 (CSI-4) for the Mexican context were conducted by three clinical psychologists with expertise in romantic relationships. Following established guidelines for test translation and cross-cultural adaptation, a back-translation procedure was conducted with the involvement of a native speaker. Subsequently, a pilot study with 50 participants was carried out to assess the clarity and comprehensibility of the translated items. Participants demonstrated adequate understanding of all four items, and no further modifications were considered necessary; therefore, the resulting version was retained for the present study. Notably, the CSI-4 has also been previously used and psychometrically examined in a Latin American population in the Dominican Republic (Díaz-Pichardo et al., 2020), providing additional support for its applicability within Spanish-speaking Latin American contexts.

Data collection occurred from July 2023 to January 2024. Participants were recruited through online advertisements, and extra credit was offered to psychology students if they invited their relatives. Participants were scheduled and invited to computer classrooms at the university. No personal information was requested during data collection; instead, participants were assigned ID numbers to ensure anonymity. All data was stored on a secure server accessible only to the principal researcher. Prior to commencement, informed consent was obtained from the participants. The survey was initiated only if they agreed to participate; otherwise, no information was collected.

After consenting to participate, the participants completed the psychological measures in the following sequence: demographic information, sexual behavior, ECR-R, CSI, NSSS-S, SAST-R, and SCL-90. On average, participants required approximately 30 to 40 min to complete all the surveys.

All participants initially completed the CSI. Participants who were currently involved in a romantic relationship responded with reference to their current relationship, whereas those without a current relationship were instructed to consider a previous significant romantic relationship. Because the CSI has primarily been validated for assessing current romantic or marital relationships, we conducted an additional sensitivity analysis excluding participants who reported no current romantic or sexual relationship (25.5% of the sample). The analyses involving couple satisfaction was repeated, and the pattern of results remained substantively unchanged. Full results of these sensitivity analyses are reported in the Supplementary material.

### 2.4 Data analysis

We computed means, standard deviations, and frequencies for all demographic characteristics within the samples to ascertain the suitable statistical tests for examining correlations and intergroup comparisons. Normality assessments were performed, encompassing the Kolmogorov-Smirnov and Shapiro-Wilk tests. Furthermore, Levene's test was employed to assess the homogeneity of variances across the five dependent variables. The results of these tests indicated that nonparametric tests would be more appropriate for the data. In order to facilitate comparison among the scales in the figures, the z-scores were calculated based on the mean and standard deviation of all participants. We utilized the latest version of G^*^Power software, version 3.1.9.7, to determine the sample size required for a linear multiple regression analysis with α = 0.05, β = 0.8, and five predictors. The current sample size (*n* = 214) aligns with the analysis conducted using G^*^Power.

Pearson correlations were used among the CSI-4, ECR-8, NSSS-S, SAST-R, and SCL-90 subscales. Additionally, in our examination of sexual behavior, we considered the correlation with the following variables: age at first sexual intercourse, number of sexual partners, contraceptive use, alcohol use during sex, and drug use during sex.

We decided to explore differences between men and women in our variables of interest: psychological distress, sexual satisfaction, attachment, couple satisfaction, and sexual behavior. We used the Mann–Whitney *U* test to compare the groups.

To compare hypersexuality and non-hypersexuality across various variables (psychological distress, sexual satisfaction, attachment, couple satisfaction, and sexual behavior), we created a categorical variable. This variable distinguishes between scores of five or lower, indicating non-hypersexuality, and scores above five, indicating hypersexuality. This 5-point cut-off is consistent with previous research using the scale in Mexico, Poland, and the United States (Mejía Cruz et al., 2023; Gola et al., 2016; Carnes et al., 2010). The Mann–Whitney U test was performed to compare the differences between the hypersexuality and non-hypersexuality groups concerning the dependent variables.

To explore variables associated with the classification of SAST-R scores, we employed a Classification and Regression Tree (CART) algorithm alongside logistic regression analysis. Given the cross-sectional design and the relatively small sample size, these analyses were considered exploratory and were not intended to identify established predictors, causal mechanisms, or prospective risk factors. Rather, the variables identified by these models were interpreted as features useful for distinguishing SAST-R classification groups within the present sample. For the CART analysis, the dataset was randomly divided into training (80%) and testing (20%) subsets. To reduce model complexity, the decision tree was restricted to a maximum of three splits. Each node displayed the corresponding decision rule, Gini impurity, and number of cases. Lower Gini impurity indicates greater homogeneity in the classification of observations within a node (for further details, see Mejía et al., 2022). The classification assigned by the model was represented in the terminal nodes (leaves) of the tree. Statistical analyses were conducted using Python 3.11 and the scikit-learn library.

## 3 Results

### 3.1 Sexual behavior

In the current sample, the mean age at first sexual intercourse was 17.01 years (SD = 1.97; Mdn = 17; range = 11–22), whereas the reported mean age of the person with whom participants first had sexual intercourse was 18.41 years (SD = 3.31; Mdn = 18; range = 11–40). The mean number of sexual partners was 4.55 (SD = 8.12). Overall, 13.5% of participants reported never having engaged in sexual intercourse. Regarding the use of protection during sexual activity, 60.74% reported using protection consistently, 20.56% reported using it sometimes, and 5.14% reported not using protection. In addition, 24.29% of participants reported drug use during sexual activity, whereas 47.66% reported alcohol use during sexual encounters.

We decided to investigate whether these characteristics of sexual behavior were associated with five psychological measures. We found that the age of initiation of sexual behavior was correlated with ECR-avoidant attachment (*r* = −0.163, *p* = 0.05); NSSS-S individual (*r* = 0.233, *p* < 0.001); and psychological distress SCL-90 (*r* = 0.189, *p* < 0.05; see Table 1).

**Table 1:** Correlations among the CSI, ECR, NSSS-S, SAST-R, SCL-90, and sexual behavior.

Variables	1	2	3	4	5	6	7	8	9	10	11	12	13
1. Age	–												
2. CSI	−0.061	–											
3. ECR anxiety	−0.082	−0.078	–										
4. ECR avoidance	−0.056	**–0.356** ^***^	0.048	–									
5. NSSS individual	−0.066	**0.296** ^***^	−0.048	**–0.280** ^***^	–								
6. NSSS interpersonal	−0.000	**0.254** ^***^	−0.062	**–0.241** ^***^	**0.806** ^***^	–							
7. SAST-TR	0.112	0.071	0.061	0.013	0.037	0.037	–						
8. SCL90	0.016	−0.104	0.103	0.008	**–0.167** ^*^	−0.131	**0.149** ^*^	–					
9. Age of sexual debut	**0.286** ^***^	−0.034	0.075	**–0.163** ^*^	**0.233** ^**^	−0.087	0.029	**0.189** ^*^	–				
10.Number sexual partners	**0.218** ^**^	0.125	−0.079	0.086	0.113	0.088	**0.353** ^***^	−0.104	−0.114	–			
11. Lack of sexual protection	**0.216** ^**^	0.056	−0.002	−0.066	0.053	0.016	**0.30** ^***^	0.012	−0.035	**0.278** ^***^	–		
12. Drug use during sex	**0.211** ^**^	0.112	−0.014	0.031	**0.235** ^***^	0.129	**0.321** ^***^	0.064	−0.133	**0.369** ^***^	**0.539** ^***^	–	
13. Alcohol use during sex	**0.229** ^***^	0.105	0.075	0.014	**0.221** ^**^	**0.215** ^**^	**0.39** ^***^	0.025	**–0.186** ^*^	**0.372** ^***^	**0.579** ^***^	**0.709** ^***^	–

^*^p < 0.05; ^**^p < 0.01; ^***^p < 0.001. The bold numbers indicate significant correlation.

In our analysis of the number of sexual partners, we observed correlations with specific factors, including SAST-R (*r* = 0.353, *p* < 0.001), alcohol consumption during sexual activity (*r* = 0.372, *p* < 0.001), drug use (*r* = 0.369, *p* < 0.001), and lack of sexual protection (*r* = 0.278, *p* < 0.001). The absence of sexual protection correlates with alcohol consumption during sexual experiences (*r* = 0.579, *p* < 0.001); drug use during sexual activity (*r* = 0.539, *p* < 0.001); and scores on the SAST-R scale (*r* = 0.300, *p* < 0.001). We identified a correlation between alcohol consumption and drug use during sexual activity (*r* = 0.709, *p* < 0.001); and scores on the SAST- R with alcohol (*r* = 0.390, *p* < 0.001), and drug use (*r* = 0.321, *p* < 0.001).

In the variable of individual sexual satisfaction of the NSSS-S, we observed a correlation between the use of alcohol (*r* = 0.221, *p* = 0.001) and drugs (*r* = 0.235, *p* < 0.001) during sexual activity. Conversely, in the variable of interpersonal sexual satisfaction, only the use of alcohol showed a correlation (*r* = 0.215, *p* = 0.002). Finally, psychological distress is correlated with the SAST-R scale (*r* = 0.149, *p* = 0.029).

### 3.2 Sexual and couple satisfaction

Considering partner satisfaction and attachment variables, we noted a significant negative correlation between ECR-avoidant attachment and CSI-4 (*r* = −0.384, *p* < 0.001). Additionally, we identified a positive correlation between individual sexual satisfaction and CSI-4 (*r* = 0.296, *p* < 0.001), as well as between interpersonal sexual satisfaction and CSI-4 (*r* = 0.254, *p* < 0.001). We found no correlation among couple satisfaction, psychological distress, and hypersexuality (see Table 1). To examine whether the inclusion of participants without a current romantic or sexual relationship affected the findings involving couple satisfaction, all relevant analyses were repeated after excluding these participants (25.5% of the total sample). The pattern of results was substantively unchanged, and the principal conclusions regarding couple satisfaction were maintained. Detailed results are provided in the Supplementary material.

In terms of sexual satisfaction, we observed a negative correlation between ECR-avoidant attachment and both individual sexual satisfaction (*r* = −0.296, *p* < 0.001) and interpersonal sexual satisfaction (*r* = −0.247, *p* < 0.001). Additionally, we identified a negative correlation between psychological distress and individual sexual satisfaction (*r* = −0.167, *p* = 0.015), with a marginally significant association observed with interpersonal sexual satisfaction (*r* = −0.131, *p* = 0.055). We found no correlation between sexual satisfaction and hypersexuality.

### 3.3 Gender differences

Considering gender, we decided to contrast men and women, given that we had too few non-binary participants to include them in the analysis (see Table 2). We found significant differences in attachment and anxiety scales: men tended to have higher levels of anxious attachment, and avoidant attachment. In terms of individual sexual satisfaction, men reported higher levels than women. Regarding couple satisfaction, no differences were observed between the groups. Similarly, no differences were found on hypersexuality scale. In the psychological distress scales, women tended to report higher levels of somatization, obsession, and depressive symptoms compared to men.

**Table 2:** Comparison of means in the main variables (ECR, NSSS-S, CSI, SCL-90, SAST-R) and demographic variables between men and women.

	Men			Women					
Variables	Mean	SD	Median	Mean	SD	Median	U	*p*	*r*_rβ_
Age	21.63	5.66	19	21.95	5.55	21	4,610.5	0.08	−0.139
Income	3,316	7,862	1	2,421	5,814	0	6,096	0.06	0.138
Age of sexual debut	16.932	1.958	17	17.667	2.525	17	2,952	0.051	−0.172
Number sexual partners	4.34	5.28	2	4.87	10.36	2	5,828	0.132	0.12
ECR-anxiety	11.04	3.52	12	10.05	3.30	9	6,397	**0.015**	0.194
ECR-avoidance	16.80	3.20	17	15.17	2.96	15	6,993	**< 0.001**	0.306
NSSS-S individual	19.68	3.86	20	18.22	4.78	19	6,197	**0.050**	0.157
NSSS-S interpersonal	21.88	4.88	23	21.07	5.95	22	5,588	0.59	0.043
CSI	14.19	5.92	16	15.30	5.82	17	4,674.5	0.112	−0.127
SCL90 somatization	1.44	0.71	1.333	1.68	0.78	1.58	4,218	**0.008**	−0.212
SCL90 obsession	0.98	0.88	0.8	1.30	0.95	1.10	4,289	**0.013**	−0.199
SCL90 sensitivity	0.64	0.67	0.444	0.81	0.78	0.56	4,583.5	0.072	−0.144
SCL90 depression	0.90	0.85	0.692	1.09	0.80	1.00	4,416	**0.029**	−0.176
SCL90 anxiety	0.66	0.76	0.3	0.76	0.75	0.60	4,878	0.265	−0.089
SCL90 hostility	0.54	0.67	0.333	0.60	0.69	0.33	4,845.5	0.231	−0.095
SCL90 phobia	0.38	0.61	0	0.48	0.67	0.17	4,655.5	0.084	−0.131
SCL90 paranoid	0.49	0.61	0.333	0.57	0.66	0.33	4,916.5	0.297	−0.082
SCL90 psychoticism	0.52	0.56	0.3	0.54	0.52	0.40	5,004	0.412	−0.066
SASTR	2.51	3.03	2	2.17	3.15	1.00	5,926	0.172	0.106

The bold numbers indicate significant differences between the groups.

### 3.4 Hypersexual behavior

In examining the correlation between hypersexuality and attachment style, we observed no significant correlation between the SAST-R and ECR scales (all *p*-values >0.05). Although overall psychological distress was significantly associated with SAST-R scores, the magnitude of this correlation was small (*r* = −0.149, *p* = 0.029), indicating that the association was modest (see Table 3).

**Table 3:** Comparison of mean for the primary variables (CSI, ECR, NSSS-S, SAST-R, SCL-90) and demographic variables between hypersexual and non-hypersexual.

	No-Hypersexual			Hypersexual					
Variables	Mean	SD	Median	Mean	SD	Median	*U*	*p*	*r*_rβ_
ECR-anxiety	10.45	3.43	10	11.39	3.53	12	1,853	0.135	−0.187
ECR-avoidance	15.95	3.18	16.30	16.30	3.23	63.50	2,235	0.877	−0.020
NSSS-S individual	18.84	4.48	20.00	18.42	4.75	19.50	2,377	0.735	0.043
NSSS-S interpersonal	21.34	5.47	22.00	21.29	5.81	22.00	2,246	0.906	−0.015
CSI	14.63	6.13	16.50	15.46	4.33	16.50	2,246	0.906	−0.015
SCL90 somatization	1.52	0.72	1.42	1.84	0.84	1.71	1,766	0.072	−0.225
SCL90 obsession	1.09	0.89	0.90	1.46	1.14	1.30	1,878.5	0.16	−0.176
SCL90 sensitivity	0.70	0.71	0.44	0.96	0.91	0.67	1,928.5	0.217	−0.154
SCL90 depression	0.96	0.78	0.85	1.35	1.15	1.23	1,901.5	0.186	−0.166
SCL90 anxiety	0.69	0.72	0.40	0.96	0.97	0.60	1,902	0.184	−0.166
SCL90 hostility	0.52	0.58	0.33	1.01	1.17	0.50	1,910	0.191	−0.162
SCL90 phobia	0.43	0.64	0.17	0.48	0.69	0.17	2,163	0.665	−0.051
SCL90 paranoid	0.49	0.59	0.33	0.91	0.86	0.75	1,659	**0.026**	−0.272
SCL90 psychoticism	0.49	0.49	0.30	0.86	0.76	0.70	1,693.5	**0.039**	−0.257

The bold numbers indicate significant differences between the groups.

Figure 1A shows that the *z*-scores for ECR Anxious and Avoidant were higher for the hypersexual group. However, a Mann–Whitney *U* test revealed no statistically significant differences in any subscale (Table 4). Figure 1B indicates that individual and interpersonal sexual satisfaction in the hypersexual group was lower compared to the non-hypersexual group. Nonetheless, participants in the hypersexual group reported greater couple satisfaction. However, no statistically significant differences were observed (Table 3). Finally, Figure 1C presents the results of the SCL-90 subscales for both groups. Participants in the higher SAST-R group showed higher scores across all SCL-90 dimensions compared with those in the lower SAST-R group; however, statistically significant differences were observed only for paranoid ideation and psychoticism (Table 3). Specifically, the higher SAST-R group reported significantly greater paranoid ideation (*U* = 1659, *p* = 0.026, ***r*_rβ_** = −0.272) and psychoticism (*U* = 1693.5, *p* = 0.039, ***r*_rβ_** = −0.257). The corresponding rank-biserial correlations indicated small-to-moderate effect sizes, suggesting that the magnitude of these group differences was modest despite reaching statistical significance. No statistically significant differences were observed for the remaining SCL-90 dimensions.

**Figure 1:**
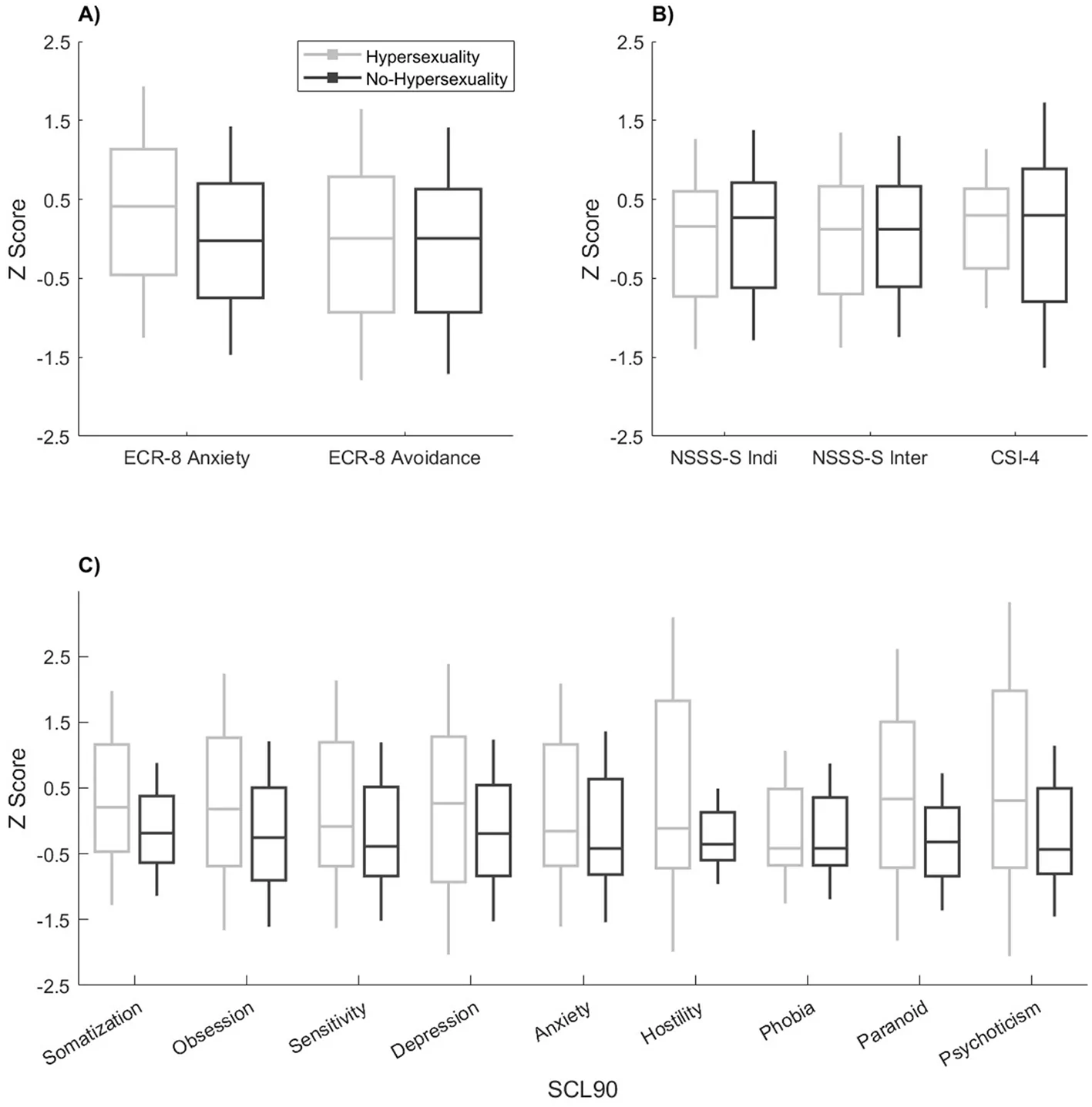
Hypersexuality and no-hypersexuality instrument scores in the variables: **(A)** ECR-8 anxiety and avoidance; **(B)** sexual satisfaction NSSS-S individual and interpersonal; **(B)** couple satisfaction CSI-16, CSI-4; **(C)** nine subscales of SCL 90, symptom checklist.

**Table 4:** Comparison of means in the sociodemographic variables and sexual behavior between hypersexual and non-hypersexual.

	No-hypersexuality		Hypersexuality				
Variables	Mean	SD	Mean	SD	*U*	*p*	*r*_rβ_
Age	21.54	5.16	23.83	8.21	1,906.00	0.19	−0.164
Income	2,834.94	6,880.28	2,734.33	6,468.33	2,192.50	0.74	−0.038
Years of education	14.03	3.28	15.57	4.02	1,690.50	0.11	−0.201
Age of sexual debut	17.289	2.312	17.56	2.55	1,707.50	0.858	0.000
Number of sexual partners	4.02	7.45	8.67	11.55	1,325.50	**0.001**	−0.406
Lack of sexual protection	2.10	0.68	2.75	0.79	1,323.50	**< 0.001**	−0.420
Alcohol use during sex	2.28	0.71	2.88	0.34	1,215.00	**< 0.001**	−0.467
Drug use during sex	2.05	0.59	2.58	0.50	1,266.00	**< 0.001**	−0.445

The bold numbers indicate significant differences between the groups.

In the held-out test sample, the CART model achieved an overall classification accuracy of 83.7%, with a sensitivity of 0.0%, specificity of 94.7%, and an area under the receiver operating characteristic curve (AUC) of 0.48. Five-fold cross-validation yielded a mean classification accuracy of 84.8% (SD = 7.0 percentage points). Although the overall accuracy and cross-validation accuracy were relatively high, these results should be interpreted cautiously because the model showed no sensitivity for identifying participants in the higher SAST-R group and an AUC close to chance-level discrimination. Thus, the relatively high overall accuracy appears to be driven primarily by the model's ability to classify participants in the lower SAST-R group rather than by balanced discrimination between the two groups.

Figure 2 presents the CART model based on standardized scores for the SCL-90 subscales. Each node displays the variable and cut-off value used to classify participants, together with the number of cases and the corresponding Gini impurity. Lower Gini values, approaching zero, indicate greater homogeneity of the observations within a node with respect to group classification. Within the exploratory tree, paranoid ideation was selected for the first split, followed by psychoticism and interpersonal sensitivity in subsequent nodes. Specifically, a standardized paranoid ideation score greater than 2.24 identified a subgroup containing participants classified in the higher SAST-R group. Among the remaining cases, standardized psychoticism scores greater than 1.88 and interpersonal sensitivity scores lower than 1.87 contributed to further classification of participants in the higher SAST-R group. Conversely, paranoid ideation scores below 2.24 combined with interpersonal sensitivity scores above 1.87 identified a terminal node composed of participants in the lower SAST-R group (Gini = 0). Although these splits indicate that paranoid ideation, psychoticism, and interpersonal sensitivity contributed to classification within the fitted tree, the poor sensitivity and AUC observed in the held-out test sample indicate limited out-of-sample discrimination of higher SAST-R cases. Accordingly, these variables should be regarded as exploratory classification features within the present sample rather than established predictors of hypersexuality-related symptoms.

**Figure 2:**
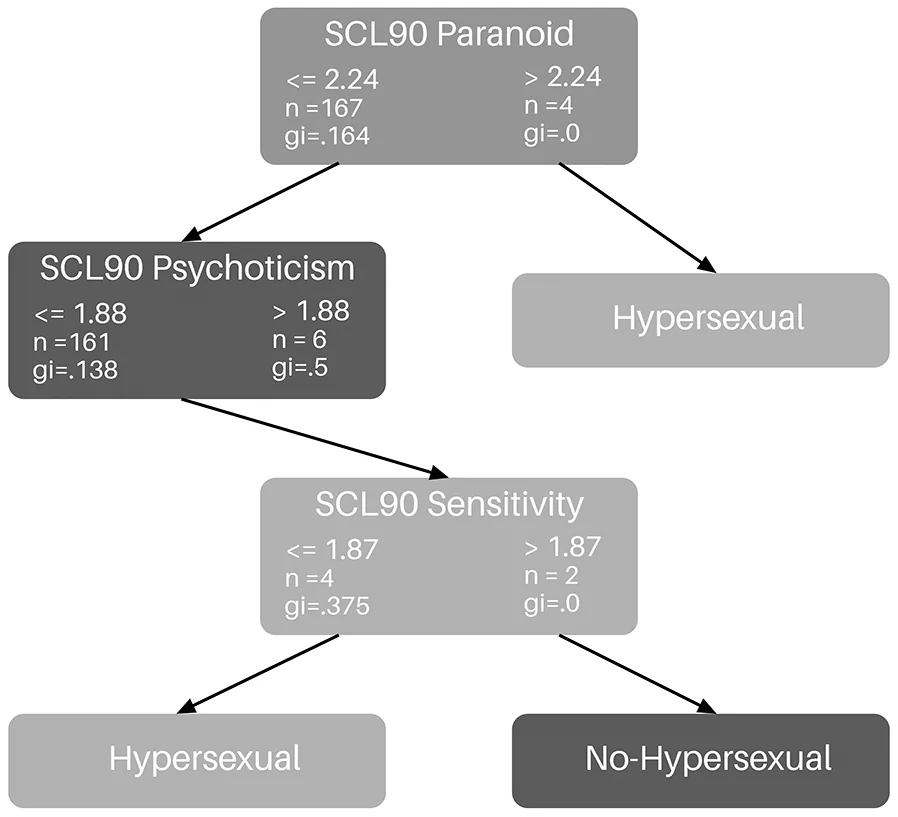
Classification and regression trees (CART) algorithm. SCL 90, symptom checklist.

In the held-out test sample, the CART model achieved an overall classification accuracy of 86.0%, with a sensitivity of 20.0%, specificity of 94.7%, and an area under the receiver operating characteristic curve (AUC) of 0.59. Five-fold cross-validation yielded a mean classification accuracy of 87.7% (SD = 0.02). Although the model demonstrated relatively high overall accuracy and specificity, its low sensitivity and modest AUC indicate limited ability to correctly identify participants in the higher SAST-R group.

Figure 3 presents the CART model examining the frequency of sexual behaviors in relation to SAST-R group classification. Within the fitted tree, frequency of drug use during sexual activity was selected for the first split, followed by number of sexual partners and frequency of sexual activity without protection in subsequent nodes. Drug use during sexual activity accounted for 44% of the higher SAST-R cases identified within the tree, whereas number of sexual partners and frequency of unprotected sexual activity were associated with 33% and 21% of these cases, respectively. Specifically, a frequency of drug use during sexual activity greater than 2.5, combined with engaging in sexual activity without protection more than 2.5 times and reporting more than 11 sexual partners, characterized a terminal node containing participants in the higher SAST-R group (Gini = 0). Conversely, lower values across these cut-off points were predominantly associated with classification into the lower SAST-R group. Although these variables contributed to classification within the fitted tree, the relatively low sensitivity (20.0%) and AUC (0.59) observed in the held-out test sample indicate that the model had limited out-of-sample ability to discriminate participants with higher SAST-R scores. Therefore, these findings should be interpreted as exploratory classification patterns within the present sample rather than evidence of established predictors of hypersexuality-related symptoms.

**Figure 3:**
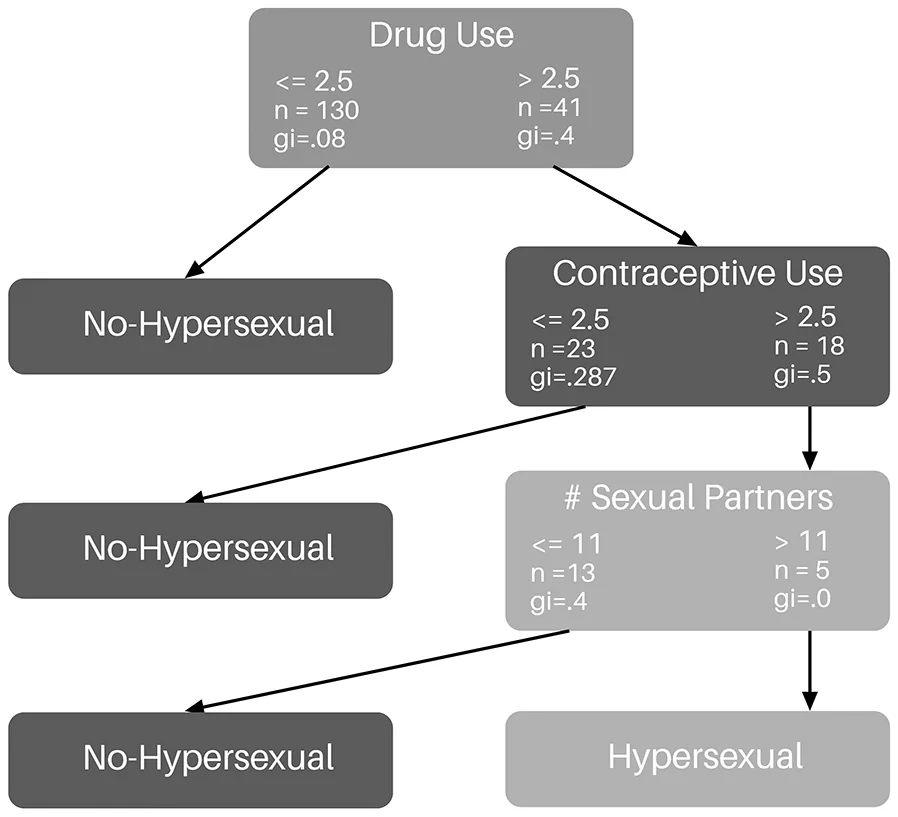
Classification and regression trees (CART) algorithm with sexual behavior.

Considering sexual behavior, Table 4 presents the socio-demographic data organized into hypersexual and non-hypersexual groups. No statistically significant differences were observed in age, income, years of education, or age at first sexual intercourse. However, differences were observed in the number of sexual partners, lack of sexual protection, alcohol use during sex, and drug use during sex. The hypersexual group tended to exhibit more risky sexual behaviors.

## 4 Discussion

In the present study, higher hypersexuality-related scores were significantly associated with several risky sexual behaviors, including a greater number of sexual partners, drug and alcohol use during sexual activity, and lack of sexual protection. This pattern is consistent with findings from previous research linking hypersexuality-related symptoms to increased engagement in risky sexual behaviors (Blanc et al., 2023; Busby et al., 2020; Gilliland et al., 2015; McElwain et al., 2015). Higher hypersexuality-related scores were also associated with greater overall psychological distress. At the group level, participants with higher SAST-R scores showed descriptively higher scores across all SCL-90 dimensions than those with lower SAST-R scores, although statistically significant differences were observed only for paranoid ideation and psychoticism. This general pattern is consistent with previous research reporting associations between hypersexuality-related symptoms and psychological distress (Ciocca et al., 2021; Dhuffar et al., 2015; Kafka, 2010; Miner et al., 2010, 2016a,b).

However, one of the main aims of the present study was to examine whether hypersexual behavior was associated with insecure attachment, as suggested by previous research (Birnbaum et al., 2014; Ciocca et al., 2021; Gilliland et al., 2015; Gori and Topino, 2024; Kotera and Rhodes, 2019; Weinstein et al., 2015; Zapf et al., 2008). Contrary to our expectations, neither attachment anxiety nor attachment avoidance was significantly associated with SAST-R scores. Thus, our findings do not support the hypothesis that attachment insecurity is directly associated with elevated hypersexuality-related symptoms in this sample. Importantly, however, the absence of an association with SAST-R scores does not imply that attachment was unrelated to participants' sexual and relational functioning. Attachment avoidance was negatively associated with both couple and sexual satisfaction, suggesting a more differentiated role of attachment in which insecurity, particularly avoidance, may be more closely related to the quality and subjective experience of intimate relationships than to hypersexual behavior itself. This distinction may help explain why previous studies have reported associations between attachment insecurity and problematic sexual outcomes while the expected relationship with hypersexuality was not observed in the present sample. It is also possible that other individual-difference processes contribute to variability in hypersexuality-related symptoms independently of attachment. For example, previous research has identified dimensions of impulsivity including urgency, lack of perseverance, lack of premeditation, and sensation seeking as potentially relevant to dysregulated sexual behavior (Cyders et al., 2007; Espinosa-Hernández et al., 2021; Reid et al., 2015). However, because impulsivity and cognitive-control processes were not assessed in the present study, they cannot be considered explanations for the current findings and should instead be examined as hypotheses in future research. Nonetheless, avoidant attachment emerged as the attachment style significantly associated with earlier sexual experiences, which may hypothetically impact sexual debut. Similar to the predictions made by Sroufe (2005) and Young et al. (2019), which suggest that secure attachment is related to the development of traits such as agreeableness and conscientiousness, fundamental elements for romantic interactions.

The present findings do not provide evidence that avoidant attachment is generally more relevant than anxious attachment to hypersexuality-related symptoms. Rather, attachment avoidance was associated with selected aspects of sexual and relational functioning, particularly lower sexual and couple satisfaction, whereas neither attachment dimension was significantly associated with SAST-R scores. This pattern suggests that the relevance of attachment may depend on the specific relational or sexual outcome under consideration. However, given the limited number and magnitude of the observed associations, these findings should be considered preliminary and require replication in larger and more diverse samples.

In the present sample, age at sexual debut was significantly and positively correlated with individual sexual satisfaction and psychological distress. Previous research has identified experiences of psychological and sexual abuse as potential developmental antecedents associated with hypersexuality and other difficulties in sexual functioning (Ciocca et al., 2021; Knight and Du, 2021; Miner et al., 2010, 2016a,b). However, the present study did not comprehensively assess histories of sexual or psychological abuse, and therefore these experiences cannot be considered explanatory factors for the observed associations involving age at sexual debut. Such experiences were addressed only broadly through a single SAST-R item, which did not assess the participant's age at the time of the experience, its timing relative to sexual debut, or the specific nature and context of the event.

In this context, we aimed to develop predictive models to identify participants with elevated hypersexuality scores. The results of the CART analysis indicated that dimensions of psychological distress were among the most relevant variables for differentiating participants according to their level of hypersexuality-related symptoms. Specifically, higher levels of paranoid ideation, psychoticism, and interpersonal sensitivity contributed to reductions in entropy (i.e., classification uncertainty), thereby improving the identification of participants with elevated hypersexuality scores. These findings are broadly consistent with previous research suggesting an association between psychological distress and heightened or dysregulated sexual behavior (Birnbaum et al., 2011, 2014). However, given the cross-sectional nature of the present study, these associations should not be interpreted as evidence that psychological distress causes hypersexual behavior.

Subsequently, in a second iteration of the CART analysis, drug use, lack of protection during sexual activity, and a higher number of sexual partners emerged as variables associated with the classification of elevated hypersexuality scores. These findings are consistent with previous studies reporting associations between hypersexuality and substance use, impulsivity, psychological distress, and engagement in risky sexual behaviors (Ciocca et al., 2021; Kafka, 2010; Reid and Meyer, 2016). Rather than indicating causal or predictive relationships, the present findings suggest that these behavioral and psychological characteristics may co-occur among individuals reporting higher levels of hypersexuality. Previous research has also linked risky sexual behaviors and substance use with increased vulnerability to adverse sexual-health outcomes, including HIV and other sexually transmitted infections, while cognitive difficulties have been documented in some populations affected by these conditions (Schuster and Gonzalez, 2012). Nevertheless, these potential pathways were not directly assessed in the present study and should not be interpreted as mechanisms underlying the observed associations.

Although the differences did not reach statistical significance, participants with elevated hypersexuality scores showed descriptively higher levels of attachment anxiety and avoidance than those with lower hypersexuality scores. Participants with elevated hypersexuality scores also reported lower levels of sexual satisfaction, consistent with previous research examining the relationship between problematic sexual behavior and relational wellbeing (Lafortune et al., 2022; Gilliland et al., 2015). Participants in the higher SAST-R group had a slightly higher mean CSI score (*M* = 15.46, SD = 4.33) than participants in the lower SAST-R group (*M* = 14.63, SD = 6.13), the median CSI score was identical in both groups (Mdn = 16.50). The Mann–Whitney comparison was not statistically significant (*U* = 2246, *p* = 0.906), and the corresponding rank-biserial correlation was very small (***r*_rβ_** = −0.015), indicating a low effect. However, given the absence of statistically significant group differences and the substantial overlap in the distributions of these variables, these descriptive patterns should be interpreted cautiously and should not be considered evidence of an association between attachment insecurity and hypersexuality in the present sample. It is possible that associations among attachment, sexual functioning, and relationship satisfaction vary across developmental stages; however, this possibility should be examined in future studies, as our sample was predominantly composed of emerging adults. During this developmental period, sexual and romantic experiences may play an important role in the development of sexual wellbeing and intimate relationships. Therefore, future longitudinal research should examine how attachment patterns and early sexual experiences are associated with subsequent sexual and relationship satisfaction across the transition from emerging to later adulthood.

Regarding gender differences, our findings were consistent with previous research, indicating that women exhibited higher levels of psychological distress compared to men (Matud et al., 2020; Viertiö et al., 2021). Specifically, women displayed elevated scores on subscales measuring somatization, obsessive symptoms, and depression. In terms of sexual satisfaction, men tended to report higher levels than women. These results align with previous studies in the field (Jackson et al., 2014).

### 4.1 Limitations and future directions

A noteworthy finding in our sample was the higher prevalence of insecure attachment among men compared to women. At this juncture, we recommend that future studies consider potential cultural factors that may help elucidate gender disparities in attachment styles.

One of the primary limitations of this study is the underrepresentation of participants from the lesbian, gay, and bisexual (LGB) community. While our findings provide valuable insights into the relationships between insecure attachment and hypersexuality, the limited diversity in sexual orientation within the sample restricts the generalizability of these results, particularly with regard to LGB community. To address this limitation, we recommend increasing the sample size to include both community and clinical populations, ensuring greater representation of diverse sexual orientations. This approach would enhance the applicability of the findings and contribute to a more comprehensive understanding of the interplay between attachment and hypersexuality across different populations.

In future studies, we recommend integrating cognitive measures to further clarify the heightened risk of developing hypersexuality, such as the Iowa Gambling Task, delay discounting task, and working memory, as observed in prior research (Reid et al., 2015; Miner et al., 2016a,b). Previous research has indicated that hypersexual behavior disrupts logical cognitive processes, a phenomenon referred to as cognitive abeyance, which is part of a hypersexuality cycle similar to that observed in substance use disorders (Phillips et al., 2019; Walton et al., 2017).

From an intervention perspective, the present findings suggest that psychological distress and risky sexual behaviors may warrant greater attention than attachment-related factors when developing prevention strategies for individuals reporting elevated hypersexuality-related symptoms. In particular, interventions may benefit from addressing psychological distress alongside sexual-health risk reduction, including substance use in sexual contexts, unprotected sexual activity, and safer sexual decision making. In contrast, because attachment dimensions were not significantly associated with SAST-R scores in the present study, our findings do not provide sufficient evidence to recommend attachment-focused interventions specifically for hypersexuality-related symptoms. Nevertheless, romantic competence may represent a complementary avenue for future prevention research. Romantic competence encompasses skills involving insight, mutuality, and emotion regulation and has been associated with healthier relationship decision making, greater relationship satisfaction, and attachment security (Davila et al., 2017, 2021). Previous research has also associated higher romantic competence with lower engagement in risky sexual behaviors, including less alcohol and substance use in connection with sexual activity (Cuccì et al., 2020). Given the relevance of risky sexual behaviors in the present findings, evaluating whether romantic competence-based approaches can promote safer sexual and relational decision making may be worthwhile. However, romantic competence was not assessed in the present study, and its potential preventive value should therefore be directly evaluated in Mexican and other Latin American populations.

### 4.2 Conclusions

In conclusion, this study offers valuable insights into sexual behavior and romantic interactions within the Latino population. We provide evidence on sexual behavior among adults, highlighting the elevated risk of developing hypersexuality associated with alcohol and drug use and its impact on psychological distress.

Specifically focusing on the Mexican population, we examined the effects of risky sexual behavior, as highlighted in previous studies (Blanc et al., 2023; Busby et al., 2020; McElwain et al., 2015) and further explored its influence on psychological distress and attachment complications. Additionally, we assessed factors beyond cultural values affecting the sexuality (Espinosa-Hernández et al., 2020; Velazquez et al., 2023), including couple and sexual satisfaction. Notably, our study is one of the few to simultaneously investigate sexual satisfaction, couple satisfaction, attachment, and sexual behavior. The integration of these variables enhances the development of innovative prevention strategies aimed at promoting secure sexual interactions and fostering healthy romantic relationships.

## Data Availability

The raw data supporting the conclusions of this article will be made available by the authors, without undue reservation.
