# Supplementary material for: Adult attachment and hypersexuality: links to couple and sexual satisfaction in a Mexican sample
Source: Front Psychol. 2026 Oct 5;17:1911233. doi: 10.3389/fpsyg.2026.1911233 (PMC13677855; doi:10.3389/fpsyg.2026.1911233)
Supplement: Supplementary file 1 [file Data_Sheet_1.pdf]

Supplementary Material

Supplementary analyses of the Couple Satisfaction Index–4 (CSI-4)

The following supplementary analyses provide additional information on the factorial structure and internal consistency of the Couple Satisfaction Index–4 (CSI-4), as well as a sensitivity analysis examining whether the findings involving couple satisfaction were substantively altered after excluding participants who reported that they were not currently engaged in any form of romantic or sexual relationship (25.5% of the sample).

1. Confirmatory factor analysis of the CSI-4

A confirmatory factor analysis (CFA) was conducted to examine the factorial structure of the CSI-4 in the present sample. The hypothesized unidimensional model showed fit to the data, $\chi^2(2) = 1.275$, CFI = 1.000, TLI = 1.000, and RMSEA < .001, 90% CI [< .001, .114]. The CSI-4 also demonstrated good internal consistency (ω = .93, 95% CI [.91, .94]). These findings support the unidimensional structure and internal consistency of the CSI-4 in the present sample.

Supplementary Table S1. Confirmatory factor analysis fit indices and internal consistency for the Couple Satisfaction Index–4 (CSI-4).

Measure	χ^2	df	CFI	TLI	RMSEA [90% CI]	BIC	AIC	ω [95% CI]
CSI-4	1.275	2	1.000	1.000	< .001 [< .001, .114]	2684.634	2643.221	.93 [.91, .94]

Note. CSI-4 = Couple Satisfaction Index–4; df = degrees of freedom; CFI = comparative fit index; TLI = Tucker–Lewis index; RMSEA = root mean square error of approximation; CI = confidence interval; BIC = Bayesian information criterion; AIC = Akaike information criterion; ω = McDonald's omega.

2. Sensitivity analysis according to current relationship status

Because 25.5% of participants reported that they were not currently engaged in any form of romantic or sexual relationship, we conducted a sensitivity analysis to examine whether their inclusion influenced findings involving couple satisfaction. The analyses involving CSI-4 scores were repeated after excluding these participants. Overall, the pattern of associations was substantively unchanged in the restricted sample.

In the restricted sample, CSI-4 scores remained significantly and negatively associated with attachment avoidance ($r = -.384$, $p < .001$) and significantly and positively associated with individual sexual satisfaction ($r = .296$, $p < .001$) and interpersonal sexual satisfaction ($r = .254$, $p < .001$). Couple satisfaction was not significantly associated with attachment anxiety ($r = -.038$, $p = .581$), SAST-R scores ($r = .071$, $p = .301$), or overall psychological distress ($r = -.104$, $p = .128$). Similarly, CSI-4 scores were not significantly associated with alcohol use during sexual activity, drug use during sexual activity, number of sexual partners, or age at sexual debut, all p values > .05. These findings indicate that excluding participants without a current romantic or sexual relationship did not materially alter the principal pattern of associations involving couple satisfaction.

Supplementary Table S2. Associations between CSI-4 couple satisfaction scores and study variables after excluding participants without a current romantic or sexual relationship.

**Variable**	**Pearson's *r* with CSI-4**	***p***
Attachment anxiety	−.038	.581
Attachment avoidance	−.384	< .001
Sexual satisfaction—individual	.296	< .001
Sexual satisfaction—interpersonal	.254	< .001
SAST-R total score	.071	.301
Psychological distress (SCL-90 total)	−.104	.128
Alcohol use during sex	.105	.125
Drug use during sex	.112	.102
Number of sexual partners	.125	.070
Age of sexual debut	−.034	.657

Note. CSI-4 = Couple Satisfaction Index–4; SAST-R = Sexual Addiction Screening Test–Revised; SCL-90 = Symptom Checklist-90. Pearson correlations were calculated after excluding participants who reported that they were not currently involved in any form of romantic or sexual relationship. Sample size may vary across correlations because of missing data.

3. CSI-4 comparison between SAST-R classification groups

As an additional robustness check, the between-group comparison of CSI-4 scores was examined after excluding participants without a current romantic or sexual relationship. The Mann–Whitney test remained nonsignificant ($U = 4674.50$, $p = .112$), and the corresponding rank-biserial correlation was small ($r_{rb} = −.127$, 95% CI [−.278, .030]). This result was consistent with the full-sample analysis, in which the group difference was also nonsignificant ($U = 2246.00$, $p = .906$) with a negligible rank-biserial correlation ($r_{rb} = −.015$, 95% CI [−.255, .227]). Thus, the substantive conclusion remained unchanged: the data did not provide evidence of a meaningful difference in couple satisfaction between the SAST-R classification groups.

Supplementary Table S3. Sensitivity analysis of CSI-4 scores across SAST-R classification groups.

**Analysis**	***U***	***p***	$\mathbf{r}_{r\beta}$	**95% CI**
Full sample	2246.00	.906	−.015	[−.255, .227]
Restricted sample	4674.50	.112	−.127	[−.278, .030]

Note. The restricted sample excludes participants who reported no current romantic or sexual relationship (25.5% of the original sample). Effect sizes are rank-biserial correlations. Confidence intervals refer to the effect-size estimates.

4. Sensitivity Analysis of CSI-4 Scores by Gender and Relationship Status

To further evaluate the robustness of the findings involving couple satisfaction, CSI-4 scores were compared between women and men in both the full sample and a restricted sample excluding participants who reported that they were not currently involved in any romantic or sexual relationship. Participants identifying with non-binary gender and those who did not report gender were not included in these gender comparisons because the present analysis was specifically conducted to compare women and men.

In the full sample, CSI-4 scores did not differ significantly between women and men, the nonparametric Mann–Whitney test also indicated no significant gender difference, $U = 2246.00$, $p = .906$, $r_{rb} = -.015$, 95% CI [−.255, .227]. In the restricted sample, the difference in CSI-4 scores between women and men remained nonsignificant, $U = 4674.50$, $p = .112$, $r_{rb} = -.127$, 95% CI [−.278, .030]. Thus, restricting the analysis according to current relationship status did not change the substantive conclusion regarding gender: CSI-4 scores did not significantly differ between women and men in either analysis.
